# An Unusual Presentation of Osteomyelitis Complicated by Pathological Fracture of the Mandible: A Case Report and Literature Review

**DOI:** 10.7759/cureus.67201

**Published:** 2024-08-19

**Authors:** Bader Fatani, Njood A AlSayari, Hamad M Bakhashwain, AlJoharah K AlShathry, Haya A Alsagr, Mohammed A Bin-Salah

**Affiliations:** 1 College of Dentistry, King Saud University, Riyadh, SAU; 2 Oral and Maxillofacial Surgery, King Saud University, Riyadh, SAU

**Keywords:** reconstruction, recon plate, mandible, pathological fracture, osteomyelitis

## Abstract

Osteomyelitis is mainly caused by pyogenic organisms that spread through fractures, the bloodstream, or surgeries. It is a serious bone infection that can be either acute or chronic. It involves an inflammatory process affecting the bone and its surrounding structures. Management of osteomyelitis mainly depends on the nature of the lesion. In pathological fracture, the usual treatment is resection of the lesion with affected margins. However, the reconstruction is usually delayed until the infected bone is completely removed. In this case report, we demonstrate a case of an unusual presentation of osteomyelitis in the right body of the mandible which caused a pathological fracture following the extraction of the lower right first molar. The patient was further treated by segmental resection, coronoidectomy, and reconstruction of the defective site using a recon plate with inferior alveolar nerve preservation using the lateralization technique.

## Introduction

Osteomyelitis is a bone-destroying inflammatory disorder induced by an invading microbe. The infection can affect multiple areas of the bone, including the marrow, cortex, periosteum, and surrounding soft tissue, and can occur at all ages. Osteomyelitis can spread locally and provide access to the bone through many sources of infection such as trauma, surgery, and joint replacement [1]. Osteomyelitis of the jaws can affect both the cortical and the cancellous parts. It affects the mandible more than the maxilla and can be classified into two main categories. Acute osteomyelitis usually takes place within one month. Most cases of acute osteomyelitis occur due to odontogenic causes such as carious teeth, periodontal infection, and pulpal inflammation. These odontogenic causes may gain access to the medullary bone through the apex of the diseased tooth. Moreover, osteomyelitis may occur following tooth extraction due to remnants of the previous infection. The symptoms that may accompanied acute osteomyelitis are pain, swelling, tenderness, trismus, and hypoesthesia. 

The other category is chronic osteomyelitis which lasts more than one month and is mostly restricted to an isolated area. In chronic osteomyelitis, pain, intra- and extraoral swelling, and facial asymmetry can be seen. Occasionally, it can accompany some systemic diseases. The usual radiographic presentation of osteomyelitis on a panoramic radiograph is an ill-defined radiolucency surrounding a socket following extraction with an erosion of the lamina dura and widening of the periodontal ligaments. Pathological mandibular fractures, which are uncommon, comprise less than 2% of all mandibular fractures [2]. They occur in areas where the bone has been weakened by an underlying disease process and can result from various causes like surgery, infections, and tumors [2,3]. Previous case reports discussed osteomyelitis of the mandible as well as the manifestation of a pathological fracture, these reports demonstrated different presentations of osteomyelitis, which were further complicated by pathological fracture [4-10]. However, these cases demonstrated different treatment approaches depending on the exact individual case. In this case report, we will demonstrate an unusual presentation of osteomyelitis in the right body of the mandible which was complicated by a pathological fracture following the extraction of the lower right first molar.

## Case presentation

**Figure 1:**
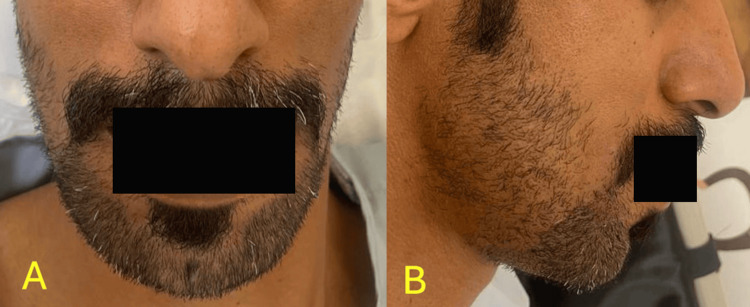
The patient's images at the time of presentation A. Frontal view of the mandible. B. Lateral view of the mandible.

A 35-year-old male patient, unaware of any medical illnesses with no known allergies came to the oral and maxillofacial surgery clinic complaining of severe pain after three weeks of extraction of a badly decayed lower right first molar. The Patient reported an increase in pain with no systematic manifestations such as fever, weight loss, or night sweats. Upon clinical examination the surgical site was intact, no active bleeding, no pus drainage, no sinus tract, or swelling were appreciated. The inferior border of the mandible showed a firm, hard, tender swelling with no step deformity detected. Extra-oral clinical photographs are presented in Figure 1. 

**Figure 2:**
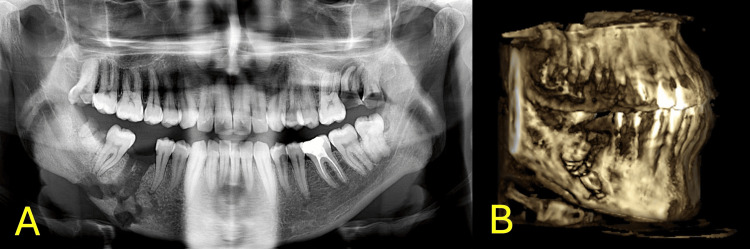
Radiographic examination images A. Pre-operative panoramic radiograph. B. Pre-operative 3-dimensional computed tomography.

The patient reported no abnormal sensation or persistent paresthesia. On radiographic examination, a pathological fracture from the extraction socket reaching the inferior border of the mandible was observed. Panoramic radiographs and CT facial bones were acquired showing irregular unclear radiolucency linked to an empty tooth socket of #46. Moreover, bone breakdown and cortical plate erosion can also be appreciated. These findings are demonstrated in Figure 2.

The patient was then prepared for an incisional biopsy of the bony lesion, which was further sent to the histopathology lab. The incisional biopsy of the bony lesion illustrated a chronic osteomyelitis causing pathological fracture of the mandible. The treatment plan was decided as segmental resection, coronoidectomy, and reconstruction of the defective site using recon plate with inferior alveolar nerve preservation using lateralization technique as well as dental extraction of hopeless teeth #18, #27, #28, #38, #45, #47 and #48. Coronoidectomy was determined in the treatment plan to avoid the effect of the temporalis muscle that pulls the coronoid process which will lead to superior rotation of the proximal segment.

**Figure 3:**
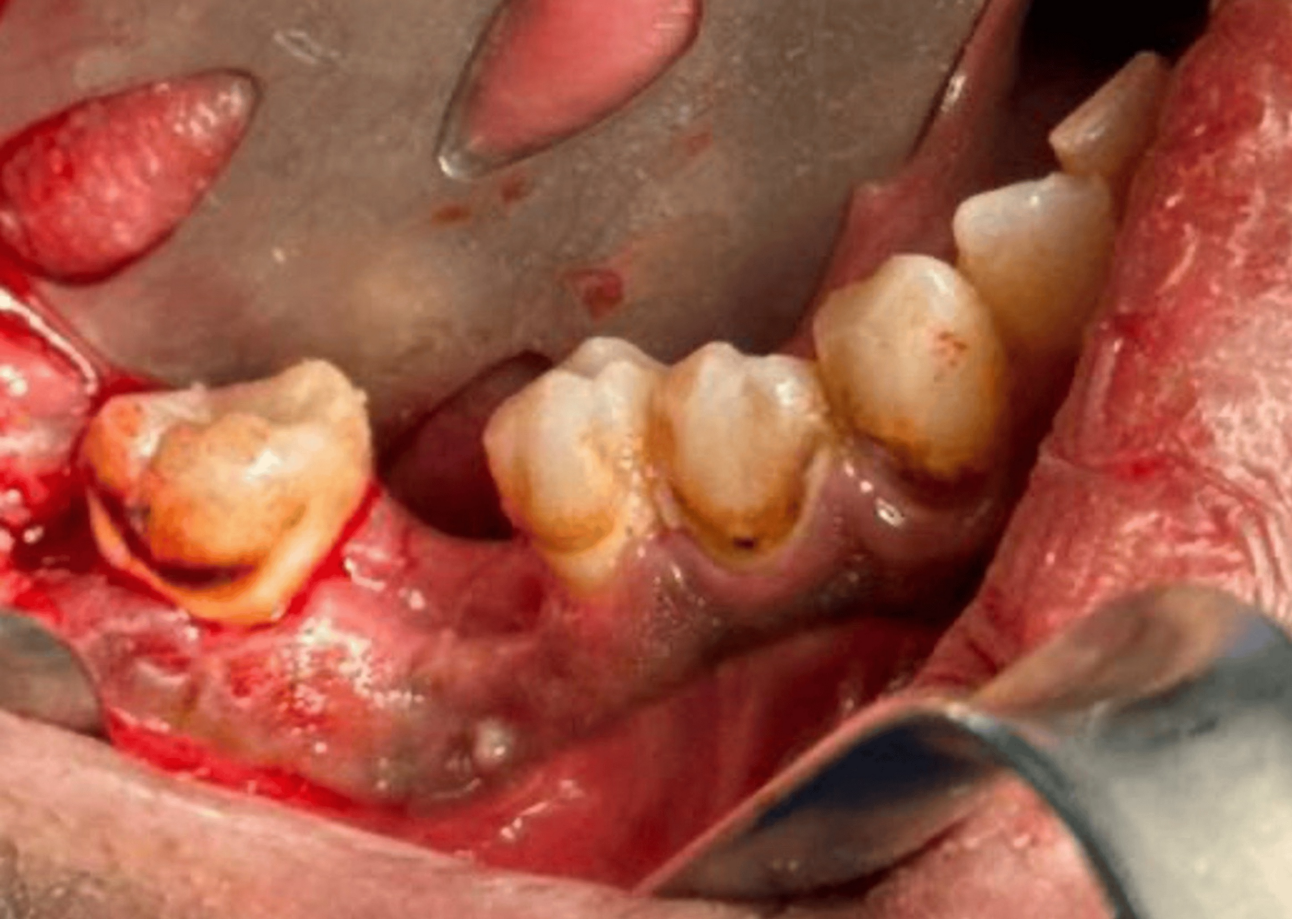
The area of the affected site before resection

The patient was brought to the operation room in the supine position, awake, conscious, and oriented. General anesthesia was induced, and the airway was secured using the right nasal intubation secured to the forehead. Figure 3 shows the area before the operation.

**Figure 4:**
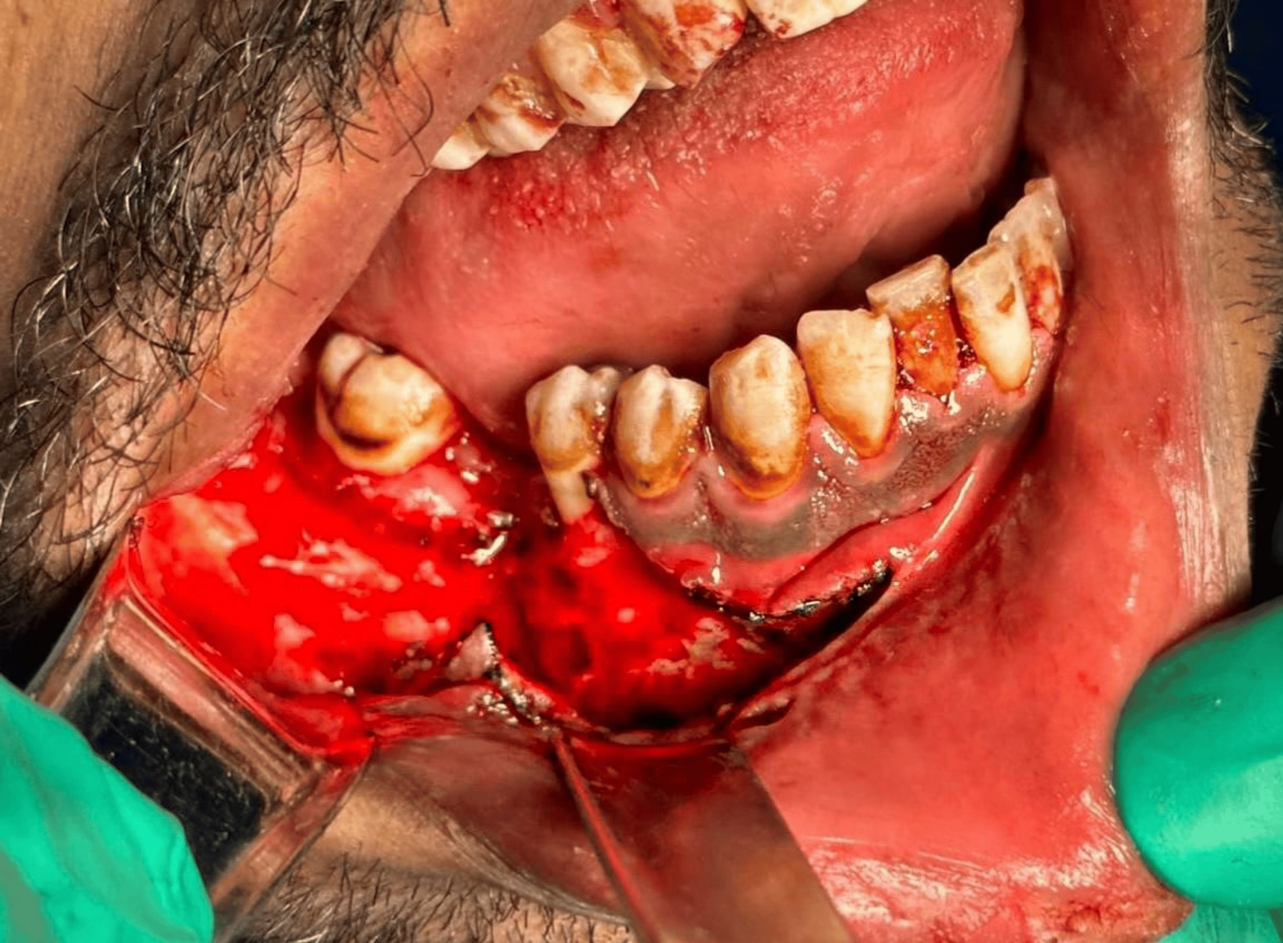
Intra-operative image The subperiosteal flap is reflected exposing the mandible from the inferior border up to the coronoid buccally and lingually.

Local anesthesia of three carpools of 2% lidocaine with 1:100,000 epinephrine was injected in all surgical sites. Dental extraction was carried out for teeth #18, 27, 28, 38, 45 and #48. The incision was then made 5 mm below mucogingival from the mandibular midline up to the area of tooth #44 with releasing incision extending the incision through the crestal mucosa posteriorly until ascending ramus area laterally. The subperiosteal flap reflected exposing the mandible from the inferior border up to the coronoid buccally and lingually in Figure 4. 

**Figure 5:**
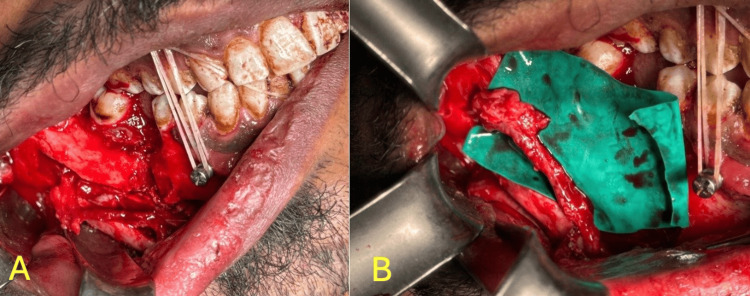
Intra-operative images A. Inferior alveolar nerve exposed by accessing through buccal bone  B. Inferior alveolar nerve lateralization.

Dissection was made up to the coronoid head followed by resection of the coronoid. Inferior alveolar nerve preservation via nerve lateralization using peizotome surgery. Figure 5 shows inferior alveolar nerve lateralization. 

**Figure 6:**
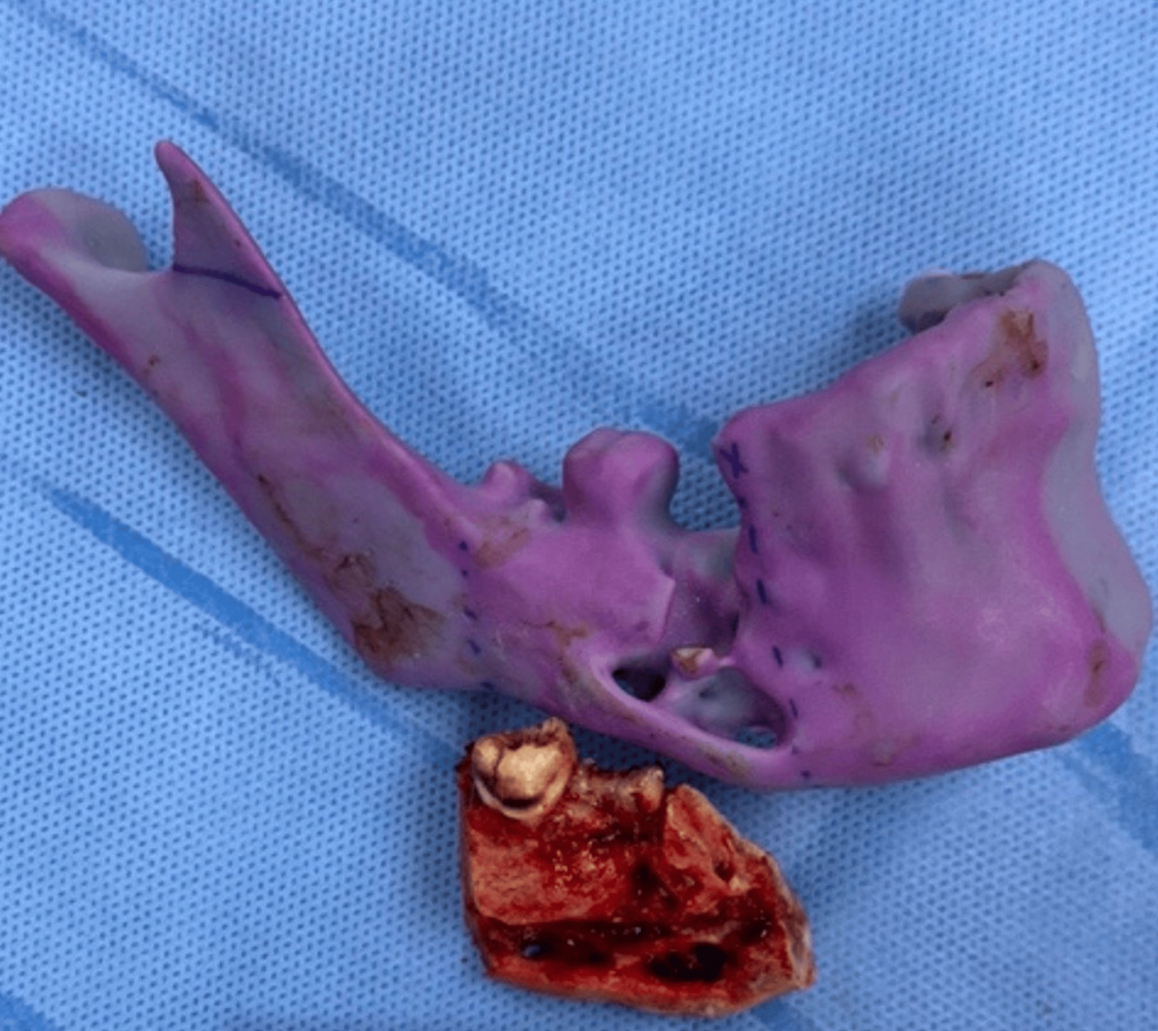
Resected area of the mandible measuring 5 cm in length

Segmental resection was prepared from the area distal to tooth #44 up to the area distal to tooth #47. Teeth #47 was included with a segmented part of the mandible and osteotomy was accomplished. The segmented area was measured at 5 cm in length and is shown in Figure 6. 

**Figure 7:**
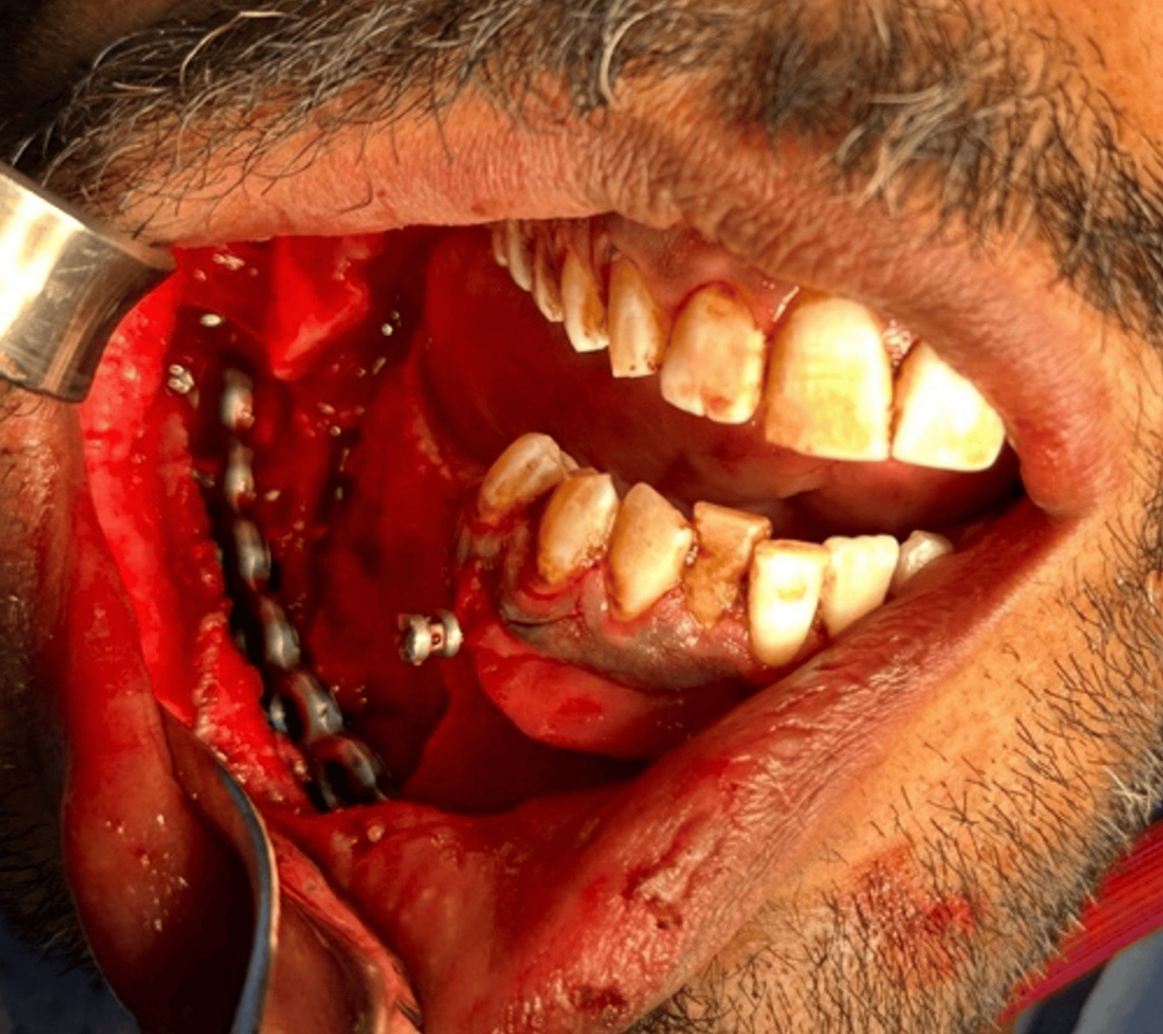
Placement of the reconstruction plate Recon plate in place after stabilization using Trochar 3 screws on each side of the mandibular edges.

Six specimens were obtained. The segmented mandible was sent for a histopathology lab, bone biopsy for acid-fast bacilli, other bone biopsies for bacterial culture, bone biopsy for fungus culture, and neuroma-like nerve tissue for a histopathology lab. The recon plate was placed and inserted using Trochar 3 screws on each side of the mandibular edges in Figure 7. 

**Figure 8:**
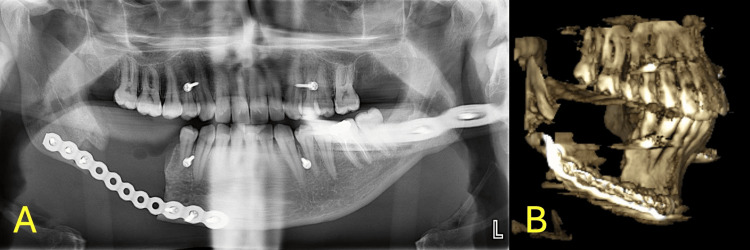
Post-operative radiographic images A. Post-operative panoramic radiograph. B. Post-operative 3-dimensional computed tomography.

The incision was irrigated with normal saline. The intra-oral incision was closed in a layered fashion using a simple interrupted technique. The incision was irrigated with normal saline. The surgical site was cleaned and dried. The patient was handed to the anesthesia team, extubated, and shifted with no complications. The patient was seen postoperatively not in pain, lying down comfortably, hemodynamically stable, sating well on room air, tolerating oral intake with no episodes of nausea or vomiting, ambulating and voiding freely, and vitally stable. The surgical site was intact, with mild oozing from the surgical site, the suture was in place, normal range of mouth opening (45 mm), mild right-sided mandibular dysesthesia, no swelling or discharge from the area, and no signs of hematoma. The postoperative panoramic and CT radiograph shows the area after resection and reconstruction using a recon plate, intermaxillary mini-screws were also placed for further stabilization of the occlusion using elastics. Panoramic and 3D CT are shown in Figure 8.

## Discussion

The mandible is the part most often affected by osteomyelitis of the jaws, which is an inflammatory process involving the cancellous and cortical portions of the bimaxillary skeleton [3]. The pathological fracture of the mandible results from an abscess eroding the bone, due to the close anatomical relationship within the same facial envelope of the parotid masseteric fascia, which includes both the mandible and the parotid gland [11]. The pathological fracture may result in some cases if osteomyelitis progresses rapidly, thus early and appropriate treatment is necessary. It is important to recognize that osteomyelitis can be caused by late fracture following third molar extraction [4]. Chronic osteomyelitis, a bone disease characterized by necrosis of mineralized and marrow tissues, suppuration, sclerosis, resorption, and hyperplasia, is primarily caused by microbiologic factors. It often results from odontogenic infections, post-extraction complications, sclerosis, inadequate removal of necrotic bone, inappropriate antibiotic selection, diagnostic failures, insufficient treatment for fractures, trauma, or irradiation of the mandible [12]. In cases of chronic osteomyelitis, bone grafting, and rigid internal fixation are necessary if there is severe bone destruction in the fractured area. For a pathological fracture caused by osteoradionecrosis, treatment involves partial resection of the mandible extending beyond the irradiated area, followed by reconstruction using free bone grafts [9]. 

This report describes an unusual presentation of osteomyelitis that was complicated by a pathological fracture, caused by extraction of a badly decayed lower right first molar. Osteomyelitis of the jaw is an inflammation that impacts both the cortical and cancellous parts of the bimaxillary skeleton. The clinical presentation, radiologic features, and treatment of mandibular osteomyelitis differ depending on the disease stage [3]. Clinical findings in mandibular osteomyelitis can include local pain, fever, swelling, purulent discharge, fistulas, paresthesia in the involved area, pathologic fracture, and trismus [13]. Our patient presented with severe pain involving his previous extraction site with a firm, hard, tender swelling in the right inferior border of the mandible. 

Pathological fractures are considered fractures that originate from normal functional or minimal disturbance in a bone weakened by any pathological process [14], they are considered rare and accounted for less than 1% in a study that included 847 fractures [15]. Late fractures after tooth extractions most likely occur during the second to fourth week [4]. The most common location where a fracture associated with osteomyelitis can be found is the angle followed by the body of the mandible [16]. The mean age was found to be 50.5 years, with a higher incidence in males [16].

Imaging helps confirm diagnoses or differentiate osteomyelitis from other abnormal pathological diseases. Conventional radiographs may not show changes immediately and can appear normal for several weeks. Panoramic X-rays indicate osteomyelitis as unclear radiolucency, often linked to an empty tooth socket or lamina dura erosion. CT scans are more precise, revealing early bone breakdown and cortical plate erosion. MRI detects inflammation in the bone marrow and soft tissues without radiation or dental filling artifacts. High-resolution CT is necessary before surgical intervention to evaluate bone damage and plan bone resection if needed [3].

Pathological fractures of the mandible are typically treated by addressing the underlying condition and stabilizing the bone fragments with osteosynthesis or arch bars and intermaxillary fixation. Treatment begins with managing systemic issues before concentrating on the specific fracture site. When feasible, free flap reconstruction should be considered [14,17]. Kim et al. [5] described a case of pathologic fracture following osteomyelitis of the mandible that was reconstructed using a fibula osteocutaneous flap following segmental resection, the author concluded that fibula osteocutaneous flap can be an adequate reconstructive treatment with good functional and aesthetic results requiring wide segmental bone resection. Coletti and Ord [14] conducted a study involving 44 cases of pathological fractures and found that the primary treatment used currently was mandibular resection of affected bone and fixation with a locking reconstruction plate only. Table 1 summarizes relevant related cases of osteomyelitis in the mandible.

**Table 1:** Review of relevant papers

Authors	Case presentation	Treatment	Conclusion
Yamamoto et al. 2019 [4].	Osteomyelitis of the mandible resulting from a delayed fracture after the extraction of a third molar.	Sequestrectomy and curettage of the defective area under general anaesthesia.	Early and proper treatment is essential when osteomyelitis of the jaw occurs due to a delayed fracture, as the condition can advance quickly and, in certain instances, lead to a pathological fracture.
Kim et al. 2021 [5].	A 76-year-old individual who had a pathologic fracture as a result of osteomyelitis in the mandible.	Reconstruction of the defected area using a fibula osteocutaneous flap following extensive segmental resection.	The main approach to managing long-standing mandibular osteomyelitis involves eliminating harmful microorganisms and removing all damaged tissues. While many cases respond well to minor resections, severe or persistent cases may require extensive resection and reconstruction, as highlighted in our research.
Barak et al. 1988 [6].	A 66-year-old woman experiencing osteoradionecrosis, a pathologic fracture, and mandibular osteomyelitis six years after undergoing radiation therapy and partial removal of the mandible due to squamous cell carcinoma of the gingiva.	The patient was treated with electromagnetic stimulation.	Nine months into the treatment, the patient showed no symptoms. Subsequent radiographic examination and bone scintigraphy at the end of the treatment confirmed the healing of osteoradionecrosis and osteomyelitis.
Ngokwe et al. 2024 [7].	A 13-year-old boy with a tooth infection had swelling on the right side of his face, difficulty opening his mouth, several pus-filled skin openings, and an underdeveloped mandible. His examination showed a fracture at the angle of the mandible, leading to the diagnosis of secondary mandibular osteomyelitis with a pathological fracture.	Sequestrectomy was performed, followed by open reduction using mini reconstruction plates.	Pathological fractures of the jaw are difficult to treat due to various causes, and clinicians frequently encounter cases with severely infected bone, with fracture treatment tailored to the resulting bone defect.
Jauhar et al. 2016 [8].	A 58-year-old female patient with chronic suppurative osteomyelitis with pathological fractures of the mandible after dental clearance.	Surgical debridement of the area. The fistulas were excised, and rigid internal fixation was applied to the mandible using a reconstruction plate.	This instance had notable factors that increased the risk of osteomyelitis and presented with distinctive clinical symptoms.
Ogasawara et al. 2008 [9].	A 43-year-old male patient with a pathological fracture of the mandible resulting from osteomyelitis	The patient was effectively treated with intermaxillary elastics.	For patients who cannot or choose not to have rigid intermaxillary fixation, this treatment option is suitable when the bone damage in the fractured area is not extensive.
Azumi et al. 1980 [10].	Two instances of mandibular fractures caused by chronic osteomyelitis are detailed.	The initial case was successfully managed with antibiotics, bone resection, and iliac bone grafting after six months of monitoring. In the second case, a diabetic patient experienced a fracture due to an Actinomyces infection spreading following the third molar extraction. Despite antibiotic treatment failure, the patient received human gamma globulin.	The inflammation signs gradually disappeared no signs of occurrence were observed.
Marschall et al. 2019 [18].	18 cases, all patients had osteomyelitis in the mandible	Patients underwent segmental removal, preservation of the inferior alveolar nerve, and immediate reconstruction using an autogenous tibia bone graft	Segmental removal of part of the jawbone is a successful approach for eliminating mandibular osteomyelitis. Additionally, prompt rebuilding using nonvascularized grafts is effective for significant defects averaging 7.1 ± 2.6 cm. However, surgery that spares the inferior alveolar nerve (IAN) is not effective in maintaining the function of the patient's IAN.
O'Sullivan et al. 2006 [19].	An uncommon outcome of peri-implantitis leading to implant failure in a 72-year-old man.	The patient received successful treatment through a rigorous oral hygiene routine, antibiotics, and conservative care.	This report emphasizes the significant personal impact of implant failure on a patient. It stresses the importance of thorough clinical and radiographic follow-up for all implant-treated patients. Such follow-ups can help detect implant failure early for timely treatment interventions.
Kato et al. 2005 [20].	A case involving pycnodysostosis with osteomyelitis and a fractured mandible.	The patient received effective treatment using a vascularized iliac bone graft.	The authors found that using a vascularized iliac bone graft is the most effective treatment for osteomyelitis and mandibular fractures in pycnodysostosis patients.
Moroni et al. 2024 [21].	A female adult with the common physical characteristics of pycnodysostosis experienced a spontaneous fracture in her right mandible at 52 years old, leading to osteonecrosis.	The patient was treated using load-bearing osteosynthesis.	This report outlines the disease progression in the patient from childhood to adulthood, emphasizing the significance of evaluating quality of life.

## Conclusions

In this paper, we demonstrate a case of osteomyelitis of the mandible that resulted in a pathological fracture which was further treated by segmental resection. It's important to be aware that a mandible fracture after extraction can lead to osteomyelitis. While many fractures are managed conservatively, prompt and proper treatment is crucial in case osteomyelitis causes a pathological fracture in the mandible, as the condition can advance quickly and may worsen in some instances. In case osteomyelitis is complicated by pathological fracture, the treatment should be determined by the extent of the lesion and the amount of affected structure. Moreover, Careful examination of the nature of the osteomyelitis and the extension of the lesion is essential in order to determine the exact approach of surgical treatment.
